# The Addition of Transarterial Chemoembolization to Palliative Chemotherapy Extends Survival in Intrahepatic Cholangiocarcinoma

**DOI:** 10.3390/jcm10122732

**Published:** 2021-06-21

**Authors:** Simon Johannes Gairing, Felix Thol, Lukas Müller, Felix Hahn, Thomas Thomaidis, Carolin Czauderna, Fabian Bartsch, Michael Bernhard Pitton, Jens Uwe Marquardt, Marcus-Alexander Wörns, Peter Robert Galle, Markus Moehler, Arndt Weinmann, Roman Kloeckner, Friedrich Foerster

**Affiliations:** 1 Department of Internal Medicine I, University Medical Center of the Johannes Gutenberg University Mainz, 55131 Mainz, Germany; simonjohannes.gairing@unimedizin-mainz.de (S.J.G.); fthol@students.uni-mainz.de (F.T.); thomaidi@uni-mainz.de (T.T.); carolin.czauderna@uksh.de (C.C.); Jens.Marquardt@uksh.de (J.U.M.); marcus-alexander.woerns@unimedizin-mainz.de (M.-A.W.), peter.galle@unimedizin-mainz.de (P.R.G.); markus.moehler@unimedizin-mainz.de (M.M.); arndt.weinmann@unimedizin-mainz.de (A.W.); 2 Department of Diagnostic and Interventional Radiology, University Medical Center of the Johannes Gutenberg University Mainz, 55131 Mainz, Germany; lukas.mueller@unimedizin-mainz.de (L.M.); felix.hahn@unimedizin-mainz.de (F.H.); michael.pitton@unimedizin-mainz.de (M.B.P.); roman.kloeckner@unimedizin-mainz.de (R.K.); 3 Department of Medicine I, University Hospital Schleswig-Holstein, 23538 Lübeck, Germany; 4 Department of General, Visceral and Transplant Surgery, University Medical Center of the Johannes Gutenberg University Mainz, 55131 Mainz, Germany; fabian.bartsch@unimedizin-mainz.de

**Keywords:** cholangiocarcinoma, chemoembolization, chemotherapy, combined modality therapy, survival

## Abstract

Incidence and mortality of intrahepatic cholangiocarcinoma (iCCA) have been increasing continuously. Recent studies suggest that the combination of palliative chemotherapy (pCTX) and transarterial chemoembolization (TACE) improves overall survival (OS). This study aimed to evaluate the outcome of patients treated with TACE and pCTX in unresectable iCCA at our tertiary care center. A group of 14 patients was treated with both pCTX and TACE. The non-randomized control group of 59 patients received pCTX alone. Patients received a median of two pCTX lines in both groups. Those treated with TACE underwent a median number of 3.5 sessions. Median OS from the time of unresectability was 26.2 months in the pCTX + TACE group versus 13.1 months in the pCTX group (*p* = 0.008). Controlling for albumin, bilirubin, ECOG (Eastern Cooperative Oncology Group) performance status, and UICC (Union for International Cancer Control) stage, the addition of TACE still conferred an OS benefit of 12.95 months (*p* = 0.014). A propensity score matching analysis yielded an OS benefit of 14 months from the time of unresectability for the pCTX + TACE group (*p* = 0.020). The addition of TACE to pCTX may provide an OS benefit for patients with unresectable iCCA. Thus, patients with liver-dominant iCCA undergoing standard-of-care pCTX should be considered for additional treatment with TACE.

## 1. Introduction

After hepatocellular carcinoma (HCC), intrahepatic cholangiocarcinoma (iCCA) represents the second most common primary malignancy of the liver [1]. Depending on localization, CCAs can be classified into intrahepatic, perihilar (pCCA), or distal tumors (dCCA). In the face of a global rise in iCCA regarding incidence and mortality, there is an urgent need for new therapeutic approaches. The majority of patients diagnosed with iCCA are not amenable to surgical resection with curative intent [2]. Both aggressive tumor biology and frequent diagnosis at an advanced stage due to the late onset of symptoms account for the poor prognosis of iCCA [3]. The main pillar of treatment for patients with unresectable CCA is palliative chemotherapy (pCTX).

PCTX with gemcitabine/cisplatin has been shown to yield a median overall survival (OS) of 11.2–11.7 months [4,5] and has become the standard-of-care first-line pCTX for patients with an ECOG (Eastern Cooperative Oncology Group) performance status (PS) of 0–1 according to the current ESMO (European Society for Medical Oncology) guidelines [6]. Individuals with a PS of 2 should rather be treated with gemcitabine monotherapy [7]. Patients with progression during treatment with gemcitabine/cisplatin should be switched to modified FOLFOX (mFOLFOX, folinic acid, fluorouracil, oxaliplatin) as second-line chemotherapy [8]. Currently, studies are evaluating the benefit of triple chemotherapy regimens such as gemcitabine/cisplatin/nab-paclitaxel or FOLFIRINOX (folinic acid, fluorouracil, irinotecan, oxaliplatin) [9,10]. Furthermore, molecularly targeted therapies such as inhibition of fibroblast growth factor receptor 1-3 (FGFR1-3) and isocitrate dehydrogenase 1 (IDH1) are being explored [11,12,13,14,15,16,17,18,19,20,21,22].

While transarterial chemoembolization (TACE) is the standard of care for patients with intermediate stage HCC [23,24,25], this is not the case for patients with iCCA. However, some evidence supporting the use of TACE in iCCA has started to emerge in patients with localized tumor burden, locoregional treatments (LRT) including TACE have shown to improve OS significantly compared to supportive therapy [26] and systemic chemotherapy [27]. The combination of LRT and systemic chemotherapy resulted in an additional survival benefit for patients with unresectable iCCA [28,29,30]. 

This single-center, retrospective study was performed to compare the outcome of patients diagnosed with unresectable iCCA receiving either systemic chemotherapy alone or in combination with TACE.

## 2. Materials and Methods

We included patients with a histologically confirmed diagnosis of unresectable iCCA, who had started pCTX at our tertiary center between January 2010 and December 2019, in our study. Patients were followed up until 31 December 2020. Patient data were collected from our institution’s electronic medical records and analyzed retrospectively. Complete blood count, blood chemistry, and the serological tumor markers carbohydrate antigen 19-9 (CA19-9) and carcinoembryonic antigen (CEA) were assessed before initiation of pCTX and during the course of treatment. After discussion in our multidisciplinary tumor board, selected patients received TACE in addition to pCTX. Response to treatment was assessed by computed tomography or magnetic resonance imaging at regular intervals. In the case of the tumor progression treatment was changed to next line chemotherapy or best supportive care.

Both patients with primarily resectable and resected iCCA and those with the primarily unresectable disease were included in this study. To improve comparability and to control for lead-time bias, the time from unresectability—besides the time from initial diagnosis—was used for OS analysis of both groups. In patients with initially resectable iCCA, the diagnosis of unresectable recurrence defined the time of unresectability. 

This study was approved by the responsible ethics committee in the state of Rhineland-Palatinate (permit number 2018-13618, clinical characterisation of the overall collective of patients with cholangiocellular carcinoma, 15 October 2018). All patients gave their written consent. The study was conducted according to the ethical guidelines of the Declaration of Helsinki 1975 and good clinical practice guidelines.

IBM SPSS Statistics version 23.0.0.3 and version 27.0.1.0 (IBM, Chicago, IL, USA) was used for statistical analysis. Regarding categorical variables, the chi-square test was used to test for statistical significance. Otherwise, an unpaired t-test was performed. To control for possible confounders, a multiple linear regression analysis was carried out. For propensity score matching, a python 3 plug-in (fuzzy, version 2.0.1, JKP IBM SPSS and PSM, version 2.0.1, JKP IBM SPSS) integrated into IBM SPSS Statistics was used. UICC (Union for International Cancer Control) stage, ECOG PS, albumin, and CA19-9 levels served as predictors for propensity score matching. The match tolerance was set to 0.4. Statistical significance was considered at *p* values < 0.05.

## 3. Results

### 3.1. Baseline Characteristics

**Figure 1:**
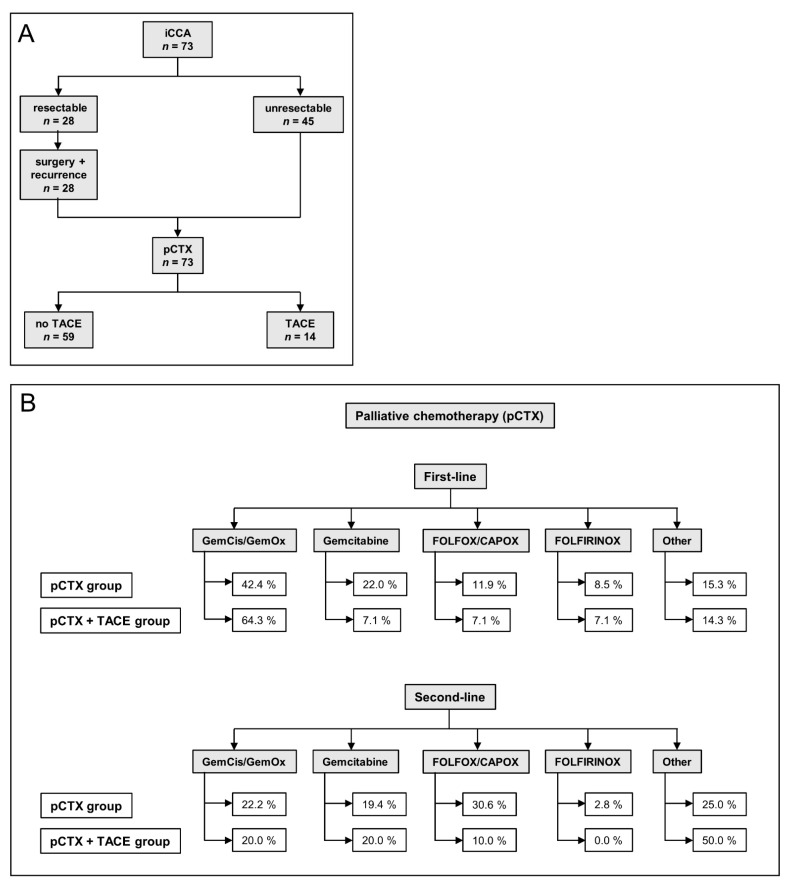
Treatment algorithm and chemotherapy regimens of the study patients. (**A**) Flow chart illustrating the treatment course of patients. iCCA: intrahepatic cholangiocarcinoma, pCTX: palliative chemotherapy, TACE: transarterial chemoembolization. (**B**) Flow chart indicating first- and second-line palliative chemotherapy (pCTX) of both groups. TACE: transarterial chemoembolization, GemCis: gemcitabine/cisplatin, GemOx: gemcitabine/oxaliplatin.

Between January 2010 and December 2019, 73 patients with a histologically confirmed diagnosis of unresectable iCCA started pCTX at our institution (Table 1). As of 31 December 2020, 63 (86.3%) patients had died (Table 2). The median follow-up was 18.1 months (range 0.9–107.5), 28 (38.4%) were initially resectable (Figure 1A). After resection, 8 patients (11.0%) received adjuvant chemotherapy. The median time from surgery to recurrence was 8.6 months. Once unresectable disease was established, 59 patients (80.8%) received pCTX alone, while 14 (19.2%) were treated with pCTX and TACE sequentially. At the time of database lock, 21.4% of patients in the pCTX + TACE group vs. 11.9% of patients in the pCTX group were still alive. 

Median age at initial diagnosis was 61.3 years in the pCTX + TACE group and 66.8 in the pCTX group (*p* = 0.073, Table 1). On average, patients in the pCTX + TACE group presented with a better ECOG PS (*p* = 0.028). Regarding gender and body mass index (BMI), no significant differences were observed. Likewise, the groups were comparable with regards to serum levels of CA19 9 and CEA prior to the administration of first line pCTX. Patients in the pCTX + TACE group showed significantly higher albumin (*p* < 0.001) and lower bilirubin (*p* = 0.007) levels prior to first-line pCTX. In terms of grading, both groups were comparable (*p* = 0.710). The UICC stage was significantly higher in the pCTX group (*p* = 0.003). 

### 3.2. Treatment

Both groups underwent a median number of two pCTX lines (Table 2). In total, 64.3% of patients in the pCTX + TACE group vs. 42.4% in the pCTX group received either gemcitabine/cisplatin or gemcitabine/oxaliplatin as first-line pCTX. A total of 7.1% of patients in the pCTX + TACE group received gemcitabine, FOLFOX/CAPOX, or FOLFIRINOX as the first line pCTX, respectively. Regarding the pCTX group, 22.0% were treated with gemcitabine, 11.9% with FOLFOX or CAPOX, and 8.5% with FOLFIRINOX (Figure 1B).

71.4% of patients in the pCTX + TACE group and 61.0% in the pCTX group received a second line pCTX after tumor progression, respectively (Table 2). The most frequent second line pCTX regimens administered in the pCTX + TACE group were gemcitabine (20.0%), gemcitabine/cisplatin or gemcitabine/oxaliplatin (20.0%) and FOLFOX or CAPOX (10.0%). Regarding the pCTX group, 30.6%, 22.2% and 19.4% received FOLFOX or CAPOX, gemcitabine/cisplatin or gemcitabine/oxaliplatin or gemcitabine, respectively. 

### 3.3. Survival

**Figure 2:**
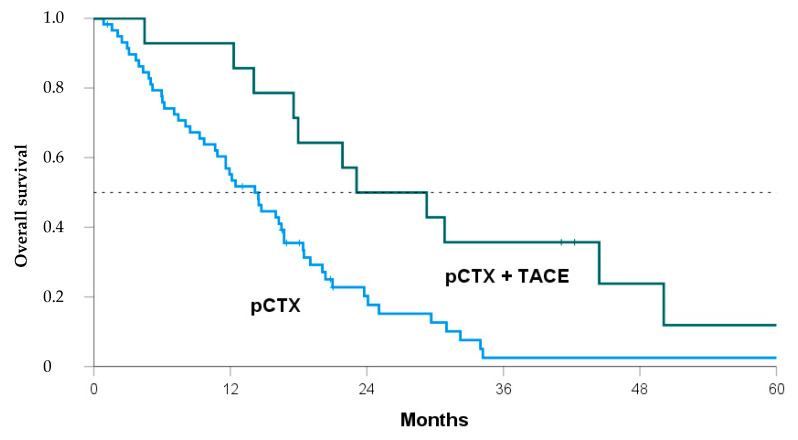
Kaplan-Meier curve comparing overall survival of patients from the time of unresectability treated with either both transarterial chemoembolization and palliative chemotherapy (pCTX + TACE, green line) or pCTX alone (blue line). Log Rank *p* = 0.005.

Median OS after diagnosis of iCCA was 30.1 months in the pCTX + TACE group vs. 17.4 months in the pCTX group (*p* = 0.019, Table 3). From the time of unresectability, the median OS was 26.2 months vs. 13.1 months (*p* = 0.008). Thus, patients receiving both TACE and pCTX showed a significant OS benefit of 12.7 months and 13.1 months, respectively. A Kaplan-Meier analysis confirmed the survival benefit for the pCTX + TACE group over the pCTX group (Figure 2, log-rank = 0.005).

To address possible confounders, a multiple linear regression analysis was performed (Supplementary Table S1). Controlling for albumin, bilirubin, ECOG PS, and UICC stage, patients receiving chemotherapy plus TACE showed a significant OS benefit of 12.95 months (*p* = 0.014). This was further confirmed by propensity score matching. Ten patients in each group showed highly comparable baseline characteristics with no significant differences regarding all tested items (Supplementary Table S2). The OS analysis still showed a significant survival benefit for the pCTX + TACE group from the time of unresectability (*p* = 0.020, Table 3).

### 3.4. Locoregional Therapy

In total, 64 TACE procedures were performed. Patients in the pCTX + TACE group received a median number of 3.5 TACE treatments (range 1–13, Table 4). Drug-eluting bead TACE (DEB-TACE) was performed in nine patients (64.3%) and conventional TACE (cTACE) in two patients (14.3%). Three patients (21.4%) received both DEB-TACE and cTACE sequentially. Half of the patients were treated with TACE before and the other half after the first administration of pCTX. No significant differences regarding OS were observed comparing the time of TACE treatment related to the start of pCTX (Supplementary Table S3). 

Patients were hospitalized for a median number of two days (range 1–26) for each TACE treatment (Table 4). In 56.3% of cases, no adverse events after LRT were observed. In 37.5%, patients suffered from postembolization syndrome including, nausea/vomiting, fever, and/or upper abdominal pain following TACE treatment. In 4.7%, severe adverse events were registered: one patient was diagnosed with a periinterventional dissection of the left hepatic artery and cholangitis. Another patient was re-hospitalized for 13 days with fever and severe deterioration of overall health. A third patient suffered from constant liver necrosis with air inclusions after TACE treatment.

### 3.5. Case Study

**Figure 3:**
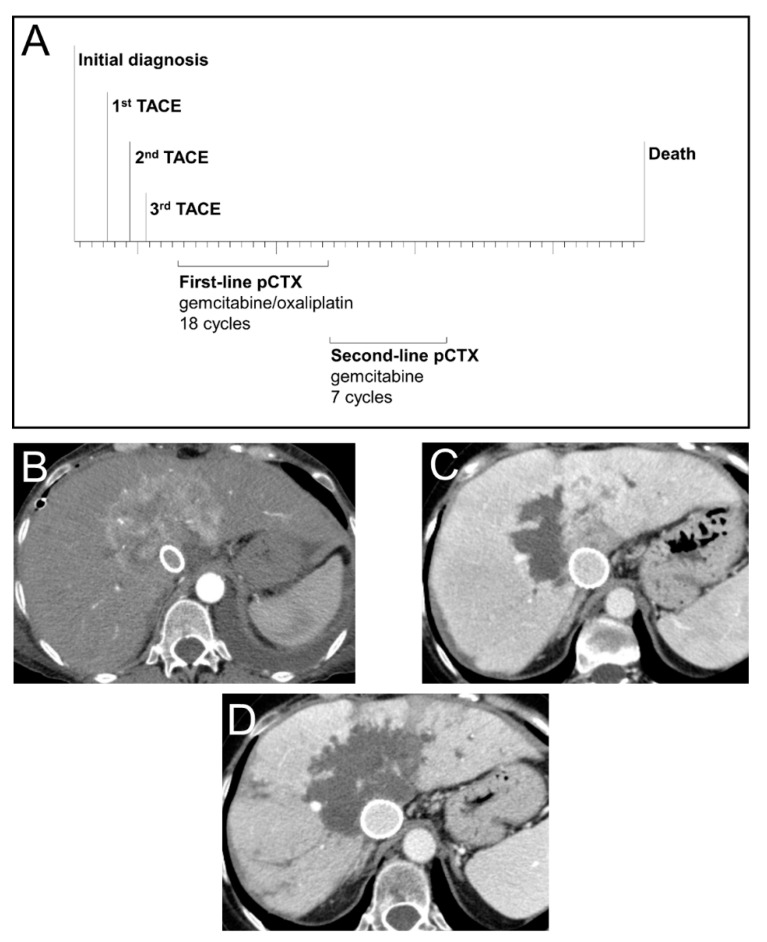
Treatment, survival and radiological response of a patient with advanced iCCA. (**A**) Time-line of sequential treatment with transarterial chemoembolization (TACE) and palliative chemotherapy (pCTX) in a 53-year-old female patient diagnosed with a centrally localized iCCA. (**B**) CT scan (arterial phase, axial) prior to first TACE treatment showing a hypervascularized large central iCCA. Due to a vena cava compression syndrome, a cava stent was implanted. (**C**) CT scan (venous phase, axial) after the first TACE treatment of the right tumour part indicating partial devascularization. (**D**) CT scan (venous phase, axial) after the third and last TACE treatment showing devascularization of the iCCA with only minimal residual tumor.

To illustrate the treatment success which can be achieved by sequential treatment with TACE and pCTX, we highlight a representative case of a 53-year-old female patient with unresectable iCCA (Figure 3). Following diagnosis, the patient was treated with three consecutive TACE sessions resulting in extensive intrahepatic tumor necrosis. The patient was then switched to pCTX and received 18 cycles of gemcitabine/oxaliplatin as first-line followed by 7 cycles of gemcitabine monotherapy as second-line treatment. This treatment sequence resulted in an OS of 50 months since diagnosis.

## 4. Discussion

In summary, the addition of TACE to pCTX resulted in a median OS benefit of 13.1 months. Both multiple linear regression and propensity score matching analyses confirmed these results. Given the dismal prognosis of unresectable iCCA, the combination of TACE and pCTX doubled the survival time of patients.

The median OS of 13.1 months from the time of unresectability reported here for patients receiving only pCTX is comparable with previous results [4,5]. Furthermore, the median OS of 26.2 months in patients who received TACE in addition to pCTX is consistent with the median OS of 28 months reported by Kiefer et al. [28].

A total of 64 TACE treatments were carried out during the follow-up period of the reported cohort. Only 3 (4.7%) severe periprocedural adverse events were registered. Thus, TACE represents a safe treatment option in patients eligible for LRT as reported previously [26,27,31,32]. A recent case series demonstrated promising survival data for the use of TACE with or without radiofrequency ablation (RFA), yielding a cumulative median OS of 29.5 months [33]. Selective internal radioembolization (SIRT) with Yttrium-90 may be an alternative locoregional approach with recently published auspicious data regarding clinical outcomes [34]. A multi-institutional study of five US centers comprising 198 patients with advanced-stage iCCA under LRT with a total of 27.8% of pCTX-pretreated individuals reported a median OS of 13.2 months. Between cTACE, DEB-TACE, bland embolization, and SIRT with Yttrium-90, no significant OS differences were found [35].

This study has some important limitations. First and foremost, the groups vary regarding ECOG PS, UICC stage, bilirubin, and albumin levels, resulting in a possible selection bias. However, the conducted multiple linear regression and propensity score matching analyses highlight that despite these differences the addition of TACE still conferred a significant survival benefit for patients eligible for TACE in our cohort. These data are in line with previously identified factors in advanced biliary cancer [36]. Second, given its retrospective design, not all baseline characteristics are available, and group sizes differ substantially (*n* = 14 vs. *n* = 59). Third, molecular profiling of iCCA including, IDH1, ARID1A, BAP1, TP53, or FGFR2 gene fusions has become part of the diagnostic routine as a basis for targeted therapy [37]. These data are not available for every patient in the study cohort.

So far, gemcitabine/cisplatin and FOLFOX have been established as pCTX protocols in the first and second lines, respectively [4,8]. A recent phase II-trial reported a median OS benefit of 2.8 months (10.1 vs. 7.3 months) for irinotecan/capecitabine (XELIRI) vs. irinotecan mono in the second line after progression on gemcitabine/cisplatin [38]. Another small retrospective cohort study demonstrated promising OS data for the combination of nanoliposomal irinotecan, 5-fluoruracil, and folinic acid in the second line setting (median OS 24.1 months) [39].

However, more effective treatments are urgently needed for patients with unresectable iCCA, but as we and others have reported not every targeted therapy is efficacious [40,41,42]. However, the FGFR1-3 inhibitor pemigatinib was recently approved for the treatment of previously treated advanced CCA with an FGFR2 fusion or other rearrangements by the FDA based on data from the FIGHT-202 trial [13]. In addition, pemigatinib is currently being evaluated as first line treatment for advanced CCA with FGFR2 rearrangement in comparison to gemcitabine/cisplatin in the FIGHT-302 phase 3 trial (NCT03656536) [11]. In parallel, other FGFR inhibitors such as derazantinib (FIDES-01 phase II trial, NCT03230318) [14,15], infigratinib (PROOF-301 phase III trial, NCT03773302) [16,17,18], Debio1347 (FUZE phase II trial, NCT03834220) [19,20] and futibatinib (FOENIX-CCA3 phase III trial, NCT04093362) [21,22] are also undergoing clinical trials. Moreover, the IDH1 inhibitor ivosidenib has been shown to improve progression-free survival in pretreated IDH1-mutated CCA in comparison to placebo in the ClarIDHy phase 3 trial (NCT02989857) [12].

Besides targeted approaches, current phase 3 trials also explore triple chemotherapy regimens with gemcitabine/cisplatin/nab-paclitaxel (NCT03768414) [9] and FOLFIRINOX [10]. While it remains to be seen what the landscape of medical treatments for CCA will exactly look like in the future, it seems obvious to test whether LRTs such as TACE can provide additional therapeutic benefit to iCCA, which might even be used in conjunction with the above-mentioned novel medical treatments.

Finally, immune checkpoint inhibitors have produced promising responses in advanced biliary tract cancer [43,44]. Furthermore, the combination of checkpoint inhibition with chemotherapy is being tested in various clinical trials (ClinicalTrials.gov Identifier: NCT03260712/NCT04066491/NCT04003636). In the future, the combination of immunotherapy with LRT such as TACE will be worthy of being explored in a clinical trial.

Given that all published data regarding LRT with TACE in unresectable iCCA were obtained in retrospective studies, a prospective, randomized, controlled and multicenter trial is needed to validate its benefit. Until then, patients with a good performance status eligible for LRT should be considered for TACE in addition to pCTX due to the promising results obtained in this and previous studies.

## Figures and Tables

**Table 1:** Baseline characteristics.

	pCTX + TACE (*n* = 14)	*n* ^†^	pCTX (*n* = 59)	*n* ^†^	*p* Value
Age at initial diagnosis—years					
median (range)	61.3 (36.7–79.3)	14	66.8 (28.8–83.1)	59	0.073
Age at first-line pCTX—years					
median (range)	61.7 (38.3–79.5)	14	67.7 (29.3–83.4)	59	0.076
Gender—no. (%)					
female	8 (57.1)	14	29 (49.2)	59	0.591
male	6 (42.9)		30 (50.8)		
BMI—kg/m^2^					
median (range)	26.4 (17.3–36.4)	13	26.0 (15.8–35.3)	53	0.332
ECOG PS at first-line pCTX—no. (%)					
0	13 (92.9)	14	24 (49.0)	49	0.028
1	1 (7.1)		19 (38.8)		
2	0 (0.0)		5 (8.5)		
3	0 (0.0)		1 (2.0)		
Initial resectability—no. (%)					
yes	4 (28.6)	14	24 (40.7)	59	0.402
Recurrence resectability—no. (%)					
yes	0 (0.0)	4	2 (8.3)	24	1.000
UICC stage—no. (%)					
1	0 (0.0)	14	7 (13.7)	51	0.003
2	10 (71.4)		10 (19.6)		
3	0 (0.0)		2 (3.9)		
4	4 (28.6)		32 (62.7)		
Grading—no. (%)					
1	0 (0.0)	7	1 (2.8)	36	0.740
2	5 (71.4)		21 (58.3)		
3	2 (28.6)		14 (38.9)		
CA19-9 (U/mL)					
median (range)	33 (4–4271)	14	66 (2–696664)	44	0.502
CEA (ng/mL)					
median (range)	1.1 (0.2–39.0)	13	1.7 (0.5–4328.1)	34	0.517
Albumin (g/L)					
median (range)	37.5 (28.0–42.0)	14	31.0 (19.0–42.0)	34	<0.001
Bilirubin (mg/dL)					
median (range)	0.7 (0.2–1.3)	14	0.9 (0.3–7.7)	49	0.007

^†^ no. of patients with available data. BMI: body mass index, ECOG PS: Eastern Cooperative Oncology Group performance status, UICC: Union for International Cancer Control, CA19-9: carbohydrate antigen 19-9, CEA: Carcinoembryonic antigen.

**Table 2:** Treatment.

	pCTX + TACE (*n* = 14)	*n* ^†^	pCTX (*n* = 59)	*n* ^†^	*p* Value
**Vital status—no. (%)**					
alive	3 (21.4)	14	7 (11.9)	59	0.392
dead	11 (78.6)		52 (88.1)		
alive-receiving pCTX treatment	1 (7.1)	14	3 (5.1)	59	1.000
**Adjuvant CTX—no. (%)**					
yes	0 (0.0)	14	8 (13.6)	59	0.340
**pCTX lines—no. (%)**					
1	4 (28.6)	14	23 (39.0)	59	0.380
2	4 (28.6)		24 (40.7)		
3	4 (28.6)		7 (11.9)		
4	2 (14.3)		4 (6.8)		
5	0 (0.0)		1 (1.7)		
median (range)	2 (1–4)		2 (1–5)		
**First-line pCTX—no. (%)**					
Gemcitabine	1 (7.1)	14	13 (22.0)	59	0.672
GemCis/GemOx	9 (64.3)		25 (42.4)		
FOLFOX/CAPOX	1 (7.1)		7 (11.9)		
FOLFIRINOX	1 (7.1)		5 (8.5)		
Other	2 (14.3)		9 (15.3)		
Cycles (median (range))	6 (2–18)	14	4 (1–27)	59	0.200
**Second-line pCTX—no. (%)**					
Gemcitabine	2 (20.0)	10	7 (19.4)	36	0.563
GemCis/GemOx	2 (20.0)		8 (22.2)		
FOLFOX/CAPOX	1 (10.0)		11 (30.6)		
FOLFIRINOX	0 (0.0)		1 (2.8)		
Other	5 (50.0)		9 (25.0)		
Cycles (median (range))	5 (1–11)	10	4 (1–16)	36	0.293
**Time surgery to recurrence—months**					
median (range)	8.3 (1.9–13.3)	4	8.6 (0.2–42.5)	24	0.550
**Time recurrence to last follow-up/death—months**					
median (range)	22.5 (18.0–44.4)	4	14.5 (1.6–79.3)	24	0.341

^†^ no. of patients with available data. pCTX: palliative chemotherapy.

**Table 3:** Overall survival.

Survival-Months	pCTX + TACE				pCTX				*p*
	*n*	Median	Q1	Q3	*n*	Median	Q1	Q3	
since initial diagnosis	14	30.1	16.7	46.8	59	17.4	6.3	25.1	0.019
since unresectability	14	26.2	16.7	42.8	59	13.1	6.1	19.0	0.008
**Survival of Propensity Score Matched Patients**									
since initial diagnosis	10	26.8	13.6	46.8	10	9.4	4.2	21.2	0.051
since unresectability	10	22.5	13.6	43.4	10	8.5	3.4	13.5	0.020

pCTX: palliative chemotherapy. TACE: transarterial chemoembolization, Q1: first quartile, Q3: third quartile.

**Table 4:** TACE treatments.

TACE Treatments	
median (range)	3.5 (1–13)
TACE method—no. (%)	
cTACE (mitomycin C)	2 (14.3)
DEB-TACE (doxorubicin)	9 (64.3)
sequential combination	3 (21.4)
Time unresectability to first TACE treatment—days	
median (range)	84.5 (13–828)
TACE/CTX relation—no. (%)	
TACE before first CTX administration	7 (50.0)
TACE after first CTX administration	7 (50.0)
Time of hospitalization per TACE treatment—days	
median (range)	2 (1–26)
Adverse events—no. (%)	
no adverse events	36 (56.3)
postembolization syndrome	24 (37.5)
severe adverse events	3 (4.7)
other	1 (1.6)

cTACE: conventional TACE, DEB-TACE: Drug-eluting bead TACE. CTX: chemotherapy.

## Data Availability

The data that support the findings of this study are available from the corresponding author, (F.F.), upon reasonable request.

## Supplementary Materials

The following are available online at https://www.mdpi.com/article/10.3390/jcm10122732/s1, Table S1: Multiple linear regression analysis, Table S2: Baseline characteristics of propensity score matched patients, Table S3: Overall survival related to start of TACE treatment.
