# Supplementary material for: The Addition of Transarterial Chemoembolization to Palliative Chemotherapy Extends Survival in Intrahepatic Cholangiocarcinoma
Source: J Clin Med. 2021 Jun 21;10(12):2732. doi: 10.3390/jcm10122732 (PMC8235389; doi:10.3390/jcm10122732)
Supplement: Supplementary file 1 [file jcm-10-02732-s001.zip › jcm-1227611-supplementary.pdf]

Supplemental files

Supplementary Table S1. Multiple linear regression analysis.

variables	regression coefficient	p
TACE	12.95	0.014
Albumin	0.92	0.026
Bilirubin	0.64	0.708
ECOG performance status	1.93	0.544
UICC stage	−1.77	0.326

Dependent variable: OS from the time of unresectability – months.

Supplementary Table S2. Baseline characteristics of propensity score matched patients.

	pCTX + TACE (n = 10)	$n^{\dagger}$	pCTX (n = 10)	$n^{\dagger}$	p value
**Age at initial diagnosis – years**					
median (range)	62.2 (43.4 – 79.3)	10	68.4 (50.2 – 73.1)	10	0.298
**Age at first-line pCTX – years**					
median (range)	63.4 (44.0 – 79.5)	10	68.6 (51.1 – 73.9)	10	0.331
**Vital status – no. (%)**					
alive	2 (20.0)	10	0 (0.0)	10	0.474
dead	8 (80.0)		10 (100.0)		
alive – receiving pCTX treatment	0 (0.0)	10	0 (0.0)	10	
**Gender – no. (%)**					
female	5 (50.0)	10	6 (60.0)	10	1.000
male	5 (50.0)		4 (40.0)		
**BMI – kg/m^2**					
median (range)	25.8 (17.3 – 36.4)	10	27.7 (15.8 – 32.4)	9	0.821
**ECOG PS at first-line pCTX – no. (%)**					
0	9 (90.0)	10	8 (80.0)	10	1.000
1	1 (10.0)		1 (10.0)		
2	0 (0.0)		1 (10.0)		
3	0 (0.0)		0		
**Initial resectability – no. (%)**					
yes	3 (30.0)	10	5 (50.0)	10	0.650
**Recurrence resectability – no. (%)**					
yes	0 (0.0)	3	1 (20.0)	5	1.000
**UICC stage – no. (%)**					
1	0 (0.0)	10	2 (20.0)	10	0.077
2	7 (70.0)		2 (20.0)		
3	0 (0.0)		1 (10.0)		
4	3 (30.0)		5 (50.0)		
**Grading – no. (%)**					
1	0 (0.0)	6	0 (0.0)	7	1.000
2	5 (83.3)		5 (71.4)		
3	1 (16.7)		2 (28.6)		
**CA19-9 (U/ml)**					
median (range)	40 (4 – 4271)	10	818 (2 – 696664)	10	0.329
**CEA (ng/ml)**					
median (range)	1.6 (0.7 – 39.0)	9	2.7 (1.6 – 312.0)	4	0.423
**Albumin (g/l)**					
median (range)	36 (28 – 41)	10	32 (21 – 40)	10	0.144

**Bilirubin (mg/dl)**					
median (range)	0.9 (0.2 – 1.3)	10	1.1 (0.3 – 5.9)	10	0.151
**Adjuvant CTX – no. (%)**					
yes	0 (0.0)	10	2 (20.0)	10	0.474
**pCTX lines – no. (%)**					
1	4 (40.0)		5 (50.0)		
2	3 (30.0)		4 (40.0)		
3	3 (30.0)	10	0 (0.0)	10	0.274
4	0 (0.0)		1 (10.0)		
5	0 (0.0)		0 (0.0)		
median (range)	2 (1 – 3)	10	1.5 (1-4)	10	
**First-line pCTX – no. (%)**					
Gemcitabine	1 (10.0)		1 (10.0)		
GemCis/GemOx	7 (70.0)		6 (60.0)		
FOLFOX/CAPOX	1 (10.0)	10	1 (10.0)	10	1.000
FOLFIRINOX	0		0 (0.0)		
Other	1 (10.0)		2 (20.0)		
**Cycles** (median (range))	4.5 (2 – 18)	10	4 (1 – 9)	10	0.247
**Second-line pCTX – no. (%)**					
Gemcitabine	1 (16.7)		1 (20.0)		
GemCis/GemOx	1 (16.7)		2 (40.0)		
FOLFOX/CAPOX	0 (0.0)	6	2 (40.0)	5	0.100
FOLFIRINOX	0 (0.0)		0 (0.0)		
Other	4 (66.7)		0 (0.0)		
**Cycles** (median (range))	3.5 (1 – 11)	6	5 (3 – 12)	5	0.182
**Time surgery to recurrence – months**					
median (range)	6.1 (1.9 – 13.3)	3	8.1 (4.7 – 14.5)	5	0.666
**Time recurrence to last follow-up/death – months**					
median (range)	21.9 (18.0 – 23.1)	3	11.6 (1.6 – 34.8)	5	0.457

[†]no. of patients with available data. CEA: Carcinoembryonic antigen, BMI: body mass index, CA19-9: carbohydrate antigen 19-9, UICC: Union for International Cancer Control, ECOG PS: Eastern Cooperative Oncology Group performance status.

Supplementary Table S3. Overall survival related to start of TACE treatment.

	**TACE before first pCTX administration**				**TACE after first pCTX administration**				
**survival (months)**	**n**	**median**	**Q1**	**Q3**	**n**	median	Q1	Q3	p
since initial diagnosis	7	30.8	14.1	54.9	7	29.3	17.6	42.3	0.475
since unresectability	7	30.8	14.1	50.1	7	23.1	17.6	41.1	0.324

pCTX: palliative chemotherapy. TACE: transarterial chemoembolization, Q1: first quartile, Q3: third quartile.
